# European CME needs the European Specialist Societies

**DOI:** 10.1080/21614083.2017.1319728

**Published:** 2017-05-04

**Authors:** Robin Stevenson, Eugene Pozniak

**Affiliations:** ^a^ Editor-in-Chief, Journal of European CME; ^b^ Managing Director, Siyemi Learning and Programme Director, European CME Forum

This is the first editorial since moving to our new publishers, Taylor & Francis. In it, we hope to make the case for developing a relationship between *JECME* and the European Specialist Societies, some of whom have contributed brief statements about their CME activities, which are appended to this paper.

The profession of medicine and those allied to it find themselves under pressure as never before. Modern doctors must be able to use complex investigative tools and be familiar with a vast array of highly potent, but also highly dangerous, drugs. They have to deal with an ageing population, much of it suffering from dementia. The working population struggles to succeed in an increasingly complex and rapidly changing environment in which physical illness is often replaced by mental illness. End-of-life care has become a minefield of competing commercial, ethical and religious interests. Doctors can exert profound influence on human life and must learn when to intervene actively and when to stand back and do nothing.

To meet the unprecedented challenges of the 21st century, today’s doctors must engage in continuing education and professional development. Cervero and Gaines [1] have convinced most sceptics that medical performance and patient outcomes are improved by CME, if it is structured appropriately. We know that needs assessment is essential, multi-media is preferable to single media, case- and workplace-based education is better that didactic lectures, repetition is important and, crucially, the education must be active rather than passive. The direction of travel in modern continuing education is best described in the seminal paper from Moore and colleagues [2] on their conceptual model for planning and assessing continuous learning for physicians.

The academic energy driving CME has largely come from North America, with Europe lagging behind. Unlike the US, few, if any, major European hospitals have dedicated CME departments. European doctors are only required to attend educational events to obtain CME credits, with no regard for performance improvement. The main providers of European CME are the European Specialist Societies, most of which have well-developed educational programmes for trainees, often including an examination. On the other hand, the provision of CME by the European Specialist Societies has traditionally been delivered with little appreciation of the major advances noted above. It is still predominantly didactic and the learning passive, based largely on the annual conference with a number of additional sub-specialty meetings.

At the 9th annual European CME Forum in Amsterdam last November, one of the sessions was devoted to the provision of CME by the European Specialist Societies. At the session, presentations were made by the European Society of Cardiology (ESC) and the International Council of Ophthalmology (ICO). The European Respiratory Society (ERS), the European Hematology Association and the European Society of Radiology were also represented. Each of these societies has made itself aware of the modern developments in CME and is in the process of changing its approach accordingly. The representatives of all the societies present found the session stimulating and challenging. Some of them admitted that they had not known about all these advances in CME, but felt that their societies would like to engage in further collaborative discussion on the subject.

It is exciting to hear that the European Specialist Societies are coming together to identify their shared responsibilities and common challenges. We hope that they will find an improved CME model for their memberships. We note that the discussion about high-quality education that addresses clinical performance and ultimately patient outcomes is not limited to the European Specialist Societies. It is encouraging that non-academic providers, educators and industry groups are also preparing their own papers in this field.

*JECME* and the European CME Forum will be hosting a virtual conference for interested European Specialist Societies devoted to these topics with a view to running a live meeting later in the year. We look forward to receiving comments from the societies on this important topic and to welcoming the contributions from other stakeholders in the European CME community.

## European Respiratory Society

Continuing professional development should be a personal learning experience, and therefore flexible for learners. ERS uses an optional and mandatory module approach to organise the curricula and training programmes. To implement the programme, ERS uses a blended learning design for continuous professional development to ensure that not only knowledge is covered, but also skills and attitudes. The curriculum is designed and activities implemented to cover a multi-disciplinary target audience.

Both the teaching and learning programmes and assessments are developed on a competency-based system that is both learner-centred and outcome-focused. It makes use of Miller’s model of clinical practice [3] and follows a two-step approach. First, it prescribes the level of competence to be achieved, aligned with prescribed learning outcomes and educational activities. Second, it addresses planning and implementing an assessment, including blueprinting, validating methods and standard setting.

## European Society of Cardiology

The ESC proposes the concept of “evidence-based, needs-driven education”, which was initially viewed with some scepticism but is now being generally accepted. It has produced world-renowned Clinical Practice Guidelines. It has also created a comprehensive eLearning platform (ESCeL) that proposes curriculum-based training programmes and a range of lifelong learning CME/continuing professional development (CPD) activities. The impacts of these educational interventions is measured by the ESC registries in association with behavioural needs assessments and outcome measurement studies.

## International Council of Ophthalmology

The ICO identifies six educational areas (CPD, teaching the teachers, accreditation and certification, team training, new educational technology, and curriculum development). This led to the formation of six committees charged with addressing these issues. In a position paper in 2014, the ICO endorsed CPD and, through its CPD Committee’s actions, aims to facilitate understanding and acceptance of the underlying concept and its promulgation and implementation (see http://www.icoph.org/resources/318/ICO-Position-Paper-in-CPD.html).

The ICO has also produced a book entitled *ICO Guide to Effective CPD/CME* with a problem-based format (www.icoph.org/ICO-CPD-CME.html).
